# A Timed Off-Switch for Dynamic Control of Gene Expression in *Corynebacterium Glutamicum*

**DOI:** 10.3389/fbioe.2021.704681

**Published:** 2021-07-29

**Authors:** Daniel Siebert, Josef Altenbuchner, Bastian Blombach

**Affiliations:** ^1^Microbial Biotechnology, Campus Straubing for Biotechnology and Sustainability, Technical University of Munich, Straubing, Germany; ^2^SynBiofoundry@TUM, Technical University of Munich, Straubing, Germany; ^3^Institute of Industrial Genetics, University of Stuttgart, Stuttgart, Germany

**Keywords:** *Corynebacterium glutamicum*, dynamic expression control, lignin, L-valine production, ferulic acid, vanillin, vanillic acid, pyruvate dehydrogenase complex

## Abstract

Dynamic control of gene expression mainly relies on inducible systems, which require supplementation of (costly) inducer molecules. In contrast, synthetic regulatory circuits, which allow the timed shutdown of gene expression, are rarely available and therefore represent highly attractive tools for metabolic engineering. To achieve this, we utilized the VanR/P_vanABK_^*^ regulatory system of *Corynebacterium glutamicum*, which consists of the transcriptional repressor VanR and a modified promoter of the *vanABK* operon (P_vanABK_^*^). VanR activity is modulated by one of the phenolic compounds ferulic acid, vanillin or vanillic acid, which are co-metabolized with d-glucose. Thus, gene expression in the presence of d-glucose is turned off if one of the effector molecules is depleted from the medium. To dynamically control the expression of the *aceE* gene, encoding the E1 subunit of the pyruvate dehydrogenase complex that is essential for growth on d-glucose, we replaced the native promoter by *vanR*/P_vanABK_^*^ yielding *C. glutamicum* ΔP_*aceE*_::*vanR*-P_vanABK_^*^. The biomass yield of this strain increased linearly with the supplemented amount of effector. After consumption of the phenolic compounds growth ceased, however, *C. glutamicum*ΔP_*aceE*_::*vanR*-P_vanABK_^*^ continued to utilize the residual d-glucose to produce significant amounts of pyruvate, l-alanine, and l-valine. Interestingly, equimolar concentrations of the three phenolic compounds resulted in different biomass yields; and with increasing effector concentration, the product spectrum shifted from pyruvate over l-alanine to l-valine. To further test the suitability of the VanR/P_vanABK_^*^ system, we overexpressed the l-valine biosynthesis genes *ilvBNCE* in *C. glutamicum* ΔP_*aceE*_::*vanR*-P_vanABK_^*^, which resulted in efficient l-valine production with a yield of about 0.36 mol l-valine per mol d-glucose. These results demonstrate that the VanR/P_vanABK_^*^ system is a valuable tool to control gene expression in *C. glutamicum* in a timed manner by the cheap and abundant phenolic compounds ferulic acid, vanillin, and vanillic acid.

## Introduction

Controlling gene expression is one of the key tasks to manipulate the metabolism of a host cell for application in a biotechnological process. Regarding the targeted induction of gene expression in prokaryotes, several systems are well-established and widely applied, such as LacI/P_*tac*_, AraC/P_*araBAD*_, PrpR/P_*prp*_, RhaR-RhaS*/*P_*rhaBAD*_, and T7 RNA polymerase-based versions (Lee and Keasling, 2005; Terpe, 2006; Brautaset et al., 2009). While these systems are usually easy to handle and well-suited for lab scale studies, their application in the industrial environment is often hampered by, e.g., the price or availability of the effector molecule (Ferreira et al., 2018; Cardoso et al., 2020). Moreover, since most effector molecules show a high stability and cannot easily be removed from the culture broth, dynamic control of gene expression is still challenging and therefore subject of recent studies in different organisms (Jayaraman et al., 2018; Baumschlager et al., 2020; Wiechert et al., 2020; Glasscock et al., 2021).

*Corynebacterium glutamicum* is a Gram-positive facultative anaerobic organism that grows on a variety of sugars, organic acids, and phenolic compounds as single or combined carbon and energy sources (Eggeling and Bott, 2005; Merkens et al., 2005; Nishimura et al., 2007; Takeno et al., 2007; Becker and Wittmann, 2019). The organism is regarded as powerhouse for large-scale production of amino acids (if mentioned here, always the l-form is meant), such as glutamate and lysine at a level of 6 million tons per year (Becker et al., 2018). Moreover, many studies exploited *C. glutamicum* for the production of commodity chemicals, such as the biofuels isobutanol, ethanol, and *n*-propanol (Inui et al., 2004; Blombach and Eikmanns, 2011; Blombach et al., 2011; Yamamoto et al., 2013; Siebert and Wendisch, 2015; Lange et al., 2018; Hasegawa et al., 2020), the diamines cadaverine and putrescine (Kind and Wittmann, 2011; Wendisch, 2017), and other amino acids such as histidine (Schwentner et al., 2019) and valine (Oldiges et al., 2014; Schwentner et al., 2018). This impressive progress in metabolic engineering of *C. glutamicum* is based on wealth of knowledge about the central metabolism, physiology, and regulation of relevant pathways, and the development of systems biology approaches and genetic engineering tools (Eggeling and Bott, 2005; Burkovski, 2008; Yukawa and Inui, 2013; Inui and Toyoda, 2020; Wang et al., 2021). Although numerous tools to manipulate the metabolism of *C. glutamicum* have been developed and standard heterologous inducible promotor systems are established and optimized (Goldbeck and Seibold, 2018; Gauttam et al., 2019), the set of available native promoters to drive synthetic and dynamic expression control is still small (Wiechert et al., 2020). Recently, Wiechert et al. (2020) constructed a GntR-dependent metabolic toggle switch that is regulated by the effector gluconate and can be applied to dynamically control gene expression in *C. glutamicum* (Wiechert et al., 2020).

**Figure 1:**
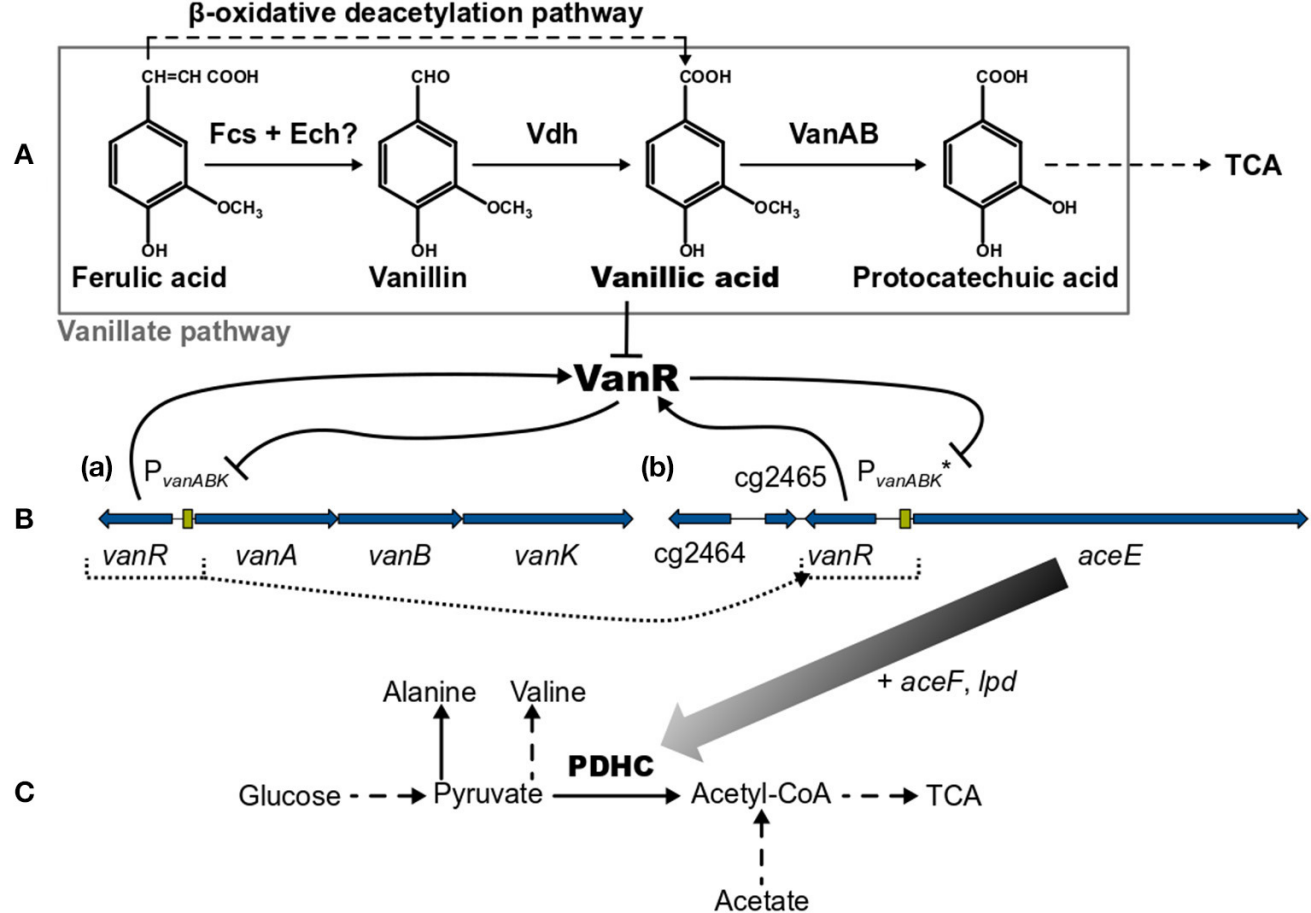
**(A)** Overview of the vanillate and β-oxidative deacetylation pathways, (**B**, a) the native *vanR*-*vanABK* gene cluster, (**B**, b) the *aceE* gene under synthetic control of VanR/P_vanABK_* (in *C. glutamicum* ΔP_*aceE*_::*vanR*-P_vanABK_*) and **(C)** relevant pathways around the pyruvate dehydrogenase complex (PDHC). Black arrows represent pathways or gene products and are dashed if indicating more than one reaction. Flat-headed lines point to inhibition. Blue arrows are genes and green boxes promotor regions. Color gradient of black arrow implies decreasing expression of *aceE* due to the utilization of phenolic compounds. Dotted lines represent the promotor exchange from P_*aceE*_ to VanR/P_vanABK_*. (?) marks that this pathway is only predicted yet. Fcs, feruloyl-CoA synthetase; Ech, enoyl-CoA hydratase/aldolase; Vdh, vanillin dehydrogenase; VanAB, A and B subunits of vanillate demethylase encoded by *vanA* and *vanB*; TCA, tricarboxylic acid cycle; VanR, PadR-type repressor encoded by *vanR*; *vanK*, gene coding for vanillate transport protein VanK; cg2464 + cg2465, genes coding for hypothetical proteins; *aceE, aceF*, and *lpd*, genes coding for E1-, E2- and E3-subunits of pyruvate dehydrogenase complex (PDHC), respectively; P_*vanABK*_, promotor region of *vanABK* and its −10-region-optimized derivative indicated by (*). This figure was adapted and compiled with information from diverse references (Kalinowski et al., 2003; Merkens et al., 2005; Brinkrolf et al., 2006; Chaudhry et al., 2007; Shen et al., 2012; Pfeifer-Sancar et al., 2013; Eikmanns and Blombach, 2014; Ding et al., 2015; Morabbi Heravi et al., 2015; Kallscheuer et al., 2016; Okai et al., 2017).

*Corynebacterium glutamicum* is able to grow on the lignin-derived phenolic compounds ferulic acid (FA), vanillin (Van), and vanillic acid (VA) and, compared with other microbial systems, is less susceptible to growth inhibition by these molecules (Shen et al., 2012; Ding et al., 2015; Becker and Wittmann, 2019). The degradation of Van and VA proceeds *via* the vanillate pathway yielding protocatechuic acid (PCA), which is subsequently converted by the β-ketoadipate pathway to acetyl-CoA and succinyl-CoA entering eventually the tricarboxylic acid (TCA) cycle [Figure 1 (Merkens et al., 2005; Brinkrolf et al., 2006; Shen et al., 2012; Kallscheuer et al., 2016; Okai et al., 2017)]. Within the vanillate pathway, FA might be converted in a cascade *via* Van and VA to PCA (Merkens et al., 2005; Brinkrolf et al., 2006; Shen et al., 2012). However, FA utilization essentially requires the action of the β-oxidative deacetylation pathway yielding VA [Figure 1 (Kallscheuer et al., 2016)]. The vanillate utilization genes are transcribed as *vanABK* operon from the promotor P_*vanABK*_, which is under control of the PadR-like repressor VanR. This transcriptional regulator is *in vitro* inhibited only by VA but not by FA, Van, or PCA; whereas *in vivo* the presence of FA, Van, and VA leads to a de-repression of the *vanABK* operon [Figure 1 (Morabbi Heravi et al., 2015)]. The crystal structure of VanR of *C. glutamicum* was recently elucidated (Yao et al., 2020) and the VanR operator sequence, which is located downstream of the P_*vanABK*_−10 region, has been identified (Morabbi Heravi et al., 2015).

In this study, we utilized the VanR/P_*vanABK*_ regulatory system to dynamically control gene expression in *C. glutamicum*. As a proof of concept, we show that the expression of the *aceE* gene, encoding the E1 subunit of the pyruvate dehydrogenase complex (PDHC), can be switched off in a timed manner, which was achieved by replacing the native *aceE* promoter with a modified VanR/P_*vanABK*_ system (subsequently indicated as VanR/P_vanABK_^*^). This dynamically controlled off-switch essentially relies on the utilization of one of the compounds, FA, Van, or VA (Figure 1), and was applied to control biomass formation and to induce the production of pyruvate and its derived products alanine and valine.

## Materials and Methods

### Microorganisms, Media, and Cultivation Conditions

All *C. glutamicum* strains and plasmids used in this study are given in Table 1. The *Escherichia coli* strain DH5α (Hanahan, 1983) was used as shuttle organism for all cloning purposes. For cultivation of *E. coli* as well as for the precultures of *C. glutamicum*, 2x TY complex medium (Green and Sambrook, 2012) was applied. The growth experiments with *C. glutamicum* were all conducted aerobically with a modified version of CGXII minimal medium (Buchholz et al., 2014) either with 5 g ammonium sulfate L^−1^, if nothing else is mentioned, or 20 g L^−1^, set to a pH of 7.4 and 20 g glucose L^−1^ as standard carbon source. Because of the phenotype of *aceE*-deficient strains (Schreiner et al., 2005), 5 g acetate L^−1^ (as potassium salt) was supplemented in precultivation steps for all *C. glutamicum* strains in this study. The standard conditions for liquid cultures were 37°C (*E. coli*) and 30°C (*C. glutamicum*) in an orbital shaker (Ø 25 mm, Multitron®2, INFORS GmbH, Einsbach, Germany) at 180 rpm applying glass tubes with 5-mL medium or 500-mL cultivation flasks with four baffles containing 50 mL medium. Solid medium was prepared by adding 18 g agar–agar L^−1^ to liquid medium in 9-cm Petri dishes, which were incubated at the above-mentioned temperature after inoculation. When appropriate, the medium was supplemented with 50 μg kanamycin mL^−1^. For the dynamic control of gene expression, FA (product no: 128708; Sigma-Aldrich Chemie GmbH, Schnelldorf, Germany), Van (product no: 7887.1; Carl Roth GmbH + Co. KG, Karlsruhe, Germany), VA (product no: H36001; Sigma-Aldrich Chemie GmbH, Schnelldorf, Germany), or protocatechuic acid (product no: B24016; Alfa Aesar by Thermo Fisher Scientific, Kandel, Germany) was dissolved in DMSO to prepare 1 M stock solutions, which were applied in appropriate dilutions to reach the final concentrations given in the results. The seed train for all cultivation experiments with *C. glutamicum* was the following: cells out of glycerol cultures (30% glycerol and stored at −80°C) were streaked out on 2x TY-agar plates, which were incubated for 3 days. A single colony was used to inoculate 2x TY medium in a glass tube, which was incubated for 6–8 h. Then, the whole suspension was transferred into 2x TY medium in cultivation flasks and incubated overnight. The culture was centrifuged (4,000 × g, 10 min, room temperature), the cells were resuspended in CGXII medium and used to inoculate the main culture (flask or FlowerPlate, see below) to a start OD_600_ (optical density at a wavelength of 600 nm) of 1. To follow the growth *via* determination of OD_600_, a spectrophotometer (ULTROSPEC® 10, Biochrom, Holliston, MA, United States) was used, diluting the samples into a range of 0.1–0.3 OD_600_ units. The cell dry weight (CDW) in gL^−1^ was calculated by the conversion factor 0.23 × OD_600_. The growth rate (h^−1^) was determined *via* linear regression in a semi-logarithmic blot by maximizing the coefficient of determination (*R*^2^) in the exponential phase. Additionally, a microbioreactor system (BioLector® I, m2p-labs GmbH, Baesweiler, Germany) for microliter scale cultivation was employed. The seed train was the same as described above but using a cultivation volume of 1 ml in a 48-well FlowerPlate (product no: MTP-48-B; m2p-labs GmbH, Baesweiler, Germany) covered with a gas permeable sealing foil (product no: F-GP-10; m2p-labs GmbH, Baesweiler, Germany). The process parameters were set to 30°C, 85% humidity, and 1,000 rpm shaking frequency. The growth was followed by measurement of the backscatter light at 620 nm with a gain of 20. In all cultivation experiments 1-mL samples were taken at the time points given in the results and centrifuged (10 min; 21300 × g) and the resulting supernatants were stored at −20°C until further analysis.

**Table 1:** Strains and plasmids used in this study.

**Strain or plasmid**	**Relevant characteristics**	**Reference or source**
***C. glutamicum*** **strains**		
WT	Wild type (ATCC13032)	Abe et al., 1967; Ikeda and Nakagawa, 2003
ΔP*_*aceE*_*::*vanR*-P_vanABK_*	Wild type with insertion of the native *vanR* gene and the optimized *vanABK* promotor sequence (indicated by asterisk, derived from pJOE7747.1) in exchange for the *aceE* promotor sequence	This study
**Plasmids**		
pK19*mobsacB*	Mobilizable *E. coli* vector for the construction of deletion and insertion mutants, Km^R^, *sacB, lacZα* with multiple cloning site, *oriV, oriT*	Schäfer et al., 1994
pK19*mobsacB*-ΔP*_*aceE*_*::*vanR*-P_vanABK_*	pK19*mobsacB*to replace the native *aceE* promotor sequence with the native *vanR* gene and the optimized *vanABK* promotor sequence (indicated by asterisk, derived from pJOE7747.1)	This study
pJOE7747.1	*ori*pBR322, *ori*pCG1, *rop*, Km^R^, *vanR*192-P*vanR*-P*vanABK*-*eGFP*-*terrrnB* (optimized−10 region of P*vanABK*)	Morabbi Heravi et al., 2015
pJC4	*E. coli*-*C. glutamicum* shuttle vector, derivative of pACYC177-pHM1519 hybrid pZ1, *ori*_p15A_, *ori*_pCG1_, Km^R^	Menkel et al., 1989; Cordes et al., 1992
pJC4-*ilvBNCE*	pJC4 with coding sequence for native *ilvBNCE* genes from *C. glutamicum* WT	Radmacher et al., 2002

### Recombinant DNA Work and Construction of *C. glutamicum* Insertion Mutant

All oligonucleotides used in this study were purchased from Sigma-Aldrich Chemie GmbH (Steinheim, Germany) and are listed in Table 2. For purification of PCR products and isolation of plasmid and genomic DNA, the kit systems “NucleoSpin® Gel and PCR Clean-up,” “NucleoSpin® Plasmid,” and “NucleoSpin® Microbial DNA” were employed, respectively, as recommended by the manufacturer MACHEREY-NAGEL GmbH & Co. KG (Düren, Germany). All enzymes were purchased from New England Biolabs GmbH [(NEB), Frankfurt am Main, Germany] and the appropriate reactions were carried out as recommended by NEB. For the construction of pK19*mobsacB*-ΔP_*aceE*_::*vanR*-P_vanABK_^*^ the flanking regions were PCR-amplified from the genomic DNA of *C. glutamicum* with the primer pairs #1 + #2 and #3 + #4 and the fragment containing the native gene *vanR* with its native promotor and the optimized *vanABK* promotor sequence from pJOE7747.1 with the primer pair #5 + #6 *via* Phusion® High-Fidelity DNA Polymerase. These fragments were cloned into SmaI-linearized pK19*mobsacB* via Gibson assembly (Gibson, 2011), preparing the assembly master mix in-house with purchased enzymes and applying the assembled plasmid for transformation of *E. coli* DH5α with the calcium chloride method (Green and Sambrook, 2012). Positive clones were identified by colony PCR using the Quick-Load® Taq 2X Master Mix adding primers #7 + #8, and the purified plasmids were further validated by restriction enzyme digestion with KpnI and sequencing of the inserted fragments *via* “TubeSeq Service” at Eurofins Genomics (Ebersberg, Germany). The plasmid pK19*mobsacB*-ΔP_*aceE*_::*vanR*-P_vanABK_^*^ was then used for transformation of electrocompetent *C. glutamicum* wild-type cells (Tauch et al., 2002) *via* electroporation (van der Rest et al., 1999). The integration of the plasmid was tested phenotypically *via* kanamycin resistance and sucrose sensitivity on 2x TY agar plates, and the whole recombination procedure *via* double cross-over events was carried out as described previously (Schäfer et al., 1994). The successful exchange of the *aceE* promotor sequence against the native *vanR* with its native promotor and the optimized *vanABK* promotor sequence in the *C. glutamicum* wild-type background was proven *via* colony PCR applying primer pair #12 + #13 and further confirmed by “TubeSeq Service” as described above, providing the purified product from the colony PCR for sequencing. The resulting strain *C. glutamicum*ΔP_*aceE*_::*vanR*-P_vanABK_^*^ was further modified by transformation with pJC4 (Cordes et al., 1992) or pJC4-*ilvBNCE* (Radmacher et al., 2002), confirming the presence of the appropriate plasmid *via* colony PCR with primer pair #17 + #18 or #16 + #17, respectively.

**Table 2:** Oligonucleotides used in this study.

**#**	**Name**	**Sequence (5^**′**^ → 3^**′**^)**
1	Pace_ex_upstrm_fw_pK19	*GTCGACTCTAGAGGATCCCCG*CCTCGAAAGCAACGAGTGAAATA
2	Pace_ex_upstrm_rv	*CAAAGCTGTAGACGCATGCA*GCTCACTGGGATTTCAGGTATCC
3	Pace_ex_dwnstrm_fw	*TAGTTCTTTAGGAGTTCCAT*ATGGCCGATCAAGCAAAACTTG
4	Pace_ex_dwnstrm_rv_pK19	*TGAATTCGAGCTCGGTACCC*GTGCACCATCCTGGTCCTTC
5	vanRP_fw_Pace_ex	*TACCTGAAATCCCAGTGAGC*TGCATGCGTCTACAGCTTTGATAC
6	vanRP_rv_Pace_ex	*AGTTTTGCTTGATCGGCCAT*ATGGAACTCCTAAAGAACTATATAACCAAATACCT
7	pK19lacZ_fw	ATGACCATGATTACGCCAAGCTTG
8	pK19lacZ_rv	TTAGCAGCCCTTGCGCC
9	pK19lacI_fw	TCAGTGAGCGAGGAAGCG
10	aceP_ex_fw_2	CGGAGCGTCGGCCAC
11	aceP_ex_rv	GGTGATGCGTGGCCCTG
12	vanRP_fw_Pace_ex_2	TTTTCTCATGCATACCCAATGCGTAC
13	vanRP_rv_Pace_ex_2	AGCTCGCTTGTAGGCTGC
14	vanR_fw	GTGGGCTTTGTGTGGAGTGG
15	vanR_rv	CTAAAGGGTATCGAGTAGTTTCAAGCC
16	fw_ilvC-2_seq	GAGTTCGGTGGCTACCTC
17	fw_pJC4_seq	CGATTGAAGACCGTCAAC
18	rev_pJC4_seq	GTCATCAGACCAAGGAG

*Italic letters: Overlapping sequence for Gibson assembly*.

### HPLC Measurement

In this study, the concentration of all compounds besides that of biomass were determined *via* high-performance liquid chromatography (HPLC) employing a 1260 Infinity II system (Agilent Technologies, Waldbronn, Germany). All columns were purchased from Agilent Technologies and only HPLC-grade solvents were used. In the case of glucose and pyruvate measurements, the system was equipped with a Hi-Plex H column (7.7 × 300mm, 8 μm) protected by a Hi-Plex H guard cartridge (3 × 5mm, 8 μm) hold at 50°C. The isocratic, mobile phase was 5 mM sulfuric acid (H_2_SO_4_) in water with a flow rate of 0.4 mL min^−1^. The signals were acquired *via* refractive index detector (RID) hold at 50°C. This method (Ball et al., 2011) was adapted from the manufacturer Agilent Technologies as well as the procedure for the analysis of the primary amino acids alanine and valine (Agilent Technologies, 2020). Therefore, an AdvanceBio Amino Acid Analysis (AAA) column (4.6 × 100mm, 2.7 μm) protected by an AdvanceBio AAA guard column (4.6 × 5mm, 2.7 μm) was installed and heated to 40°C. The separation was carried out *via* a gradient with an aqueous, polar phase (10mM Na_2_HPO_4_, 10mM Na_2_B_4_O7, pH 8.2) and a non-polar phase (45 vol% acetonitrile, 45 vol% methanol, 10 vol% water). For detection of the primary amino acids, an automated online derivatization with *ortho*-phthaldialdehyde (OPA) was conducted, and signals were acquired *via* fluorescence detector (FLD) at an excitation wavelength of 340 nm, an emission wavelength of 450 nm, and a PMT gain of 10. As internal standard, 100 μM norvaline was added to each sample. The measurement of the phenolic compounds FA, Van, and VA was adapted from Mollerup Andersen and Batsberg Pedersen (1983) and Merkens et al. (2005) using the same column described in the amino acids analysis. For the separation, a gradient from 95% 30 mM formic acid in water decreasing to 65% within 15 min was adjusted, applying as second phase pure methanol with an overall constant flow rate of 1 mL min^−1^. FA and Van were detected at 280 nm and VA at 264 nm in a diode-array detector (DAD). For all HPLC analysis peak detection, integration and calculation of the final concentrations were carried out with the software “OpenLab CDS ChemStation Edition Rev. C.01.10 [236]” (Agilent Technologies, Waldbronn, Germany), which was also employed for the instrument controls. For the calibration curves, external reference standards were prepared for each compound ranging from 1 to 200 mM with seven points (glucose and pyruvate), 10 to 400 μM with seven points (alanine and valine), or 0.01 to 25 mM with 11 points (phenolics). Sample preparation was carried out by thawing of the supernatants, additional centrifugation (10 min; 21300 × g) and transfer of the resulting supernatants into HPLC glass vials. The samples were diluted if appropriate.

## Results

### Influence of the Phenolic Compounds Ferulic Acid, Vanillin, and Vanillic Acid on Growth of *C. glutamicum*

**Figure 2:**
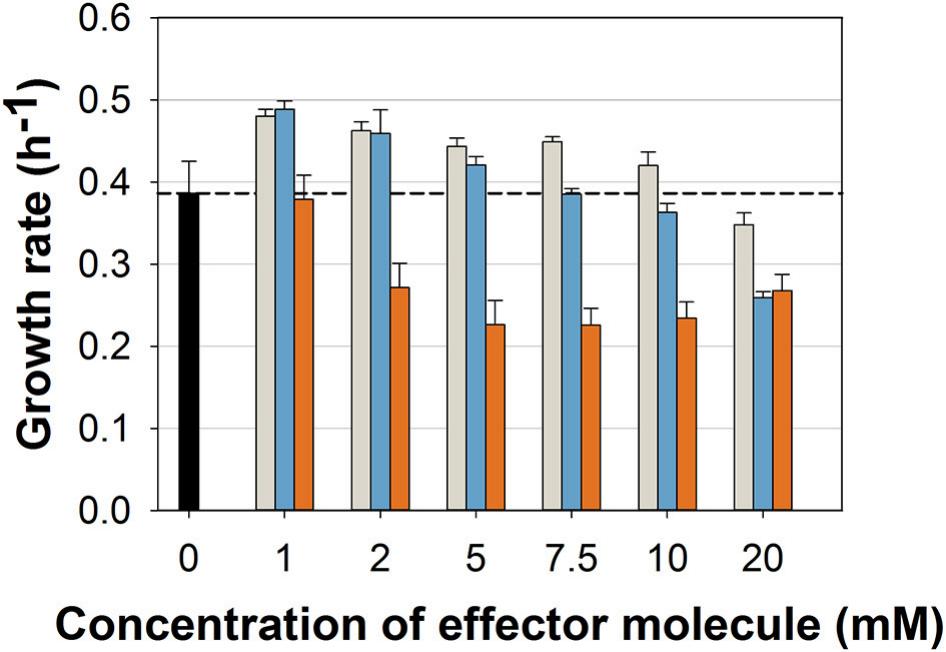
Growth rates of *C. glutamicum* wild type in microliter scale cultivations with CGXII minimal medium and 20 g glucose L^−1^ without (black) and with different concentrations of ferulic acid (gray), vanillin (blue), and vanillic acid (orange). Black dashed line indicates mean growth rate of the wild type without any effector molecule. Growth rate was determined for all cultivations between 20 and 400 backscatter units *via* linear regression in a semi-logarithmic plot (Supplementary Figure 1). Error bars represent standard deviation of at least three cultivations.

While the general ability to utilize ferulic acid, vanillin, and vanillic acid as sole carbon source for growth was already shown (Merkens et al., 2005; Brinkrolf et al., 2006), we aimed at first to investigate which concentrations of these potential antimicrobial compounds may hamper the growth of the *C. glutamicum* wild type. We cultivated the wild type strain in a microliter scale (Biolector® I, M2PLabs, Baesweiler, Germany) with the CGXII minimal medium and 20 g glucose L^−1^ supplemented with different concentrations of FA, Van, and VA. Interestingly, FA and Van at concentrations up to 10 mM did not inhibit growth (Figure 2). In contrast, low concentrations (0–5 mM) of both phenolic compounds even increased the growth rate by maximal 24 and 27%, respectively. Only at 10 and 20 mM of FA and Van growth was negatively affected. VA showed the strongest inhibitory effect with 2 mM already leading to a decrease of 30% in the growth rate. While the finally reached backscatter values were almost the same, the lag phase was prolonged with higher concentrations of the supplemented substances (Supplementary Figure 1). Based on these results, we conducted the characterization of the strain *C. glutamicum* ΔP_*aceE*_::*vanR*-P_vanABK_^*^ with concentration up to 10 mM for all the three compounds.

### Adjusting Growth of *C. glutamicum* ΔP*_*aceE*_*::*vanR*-P_vanABK_^*^ With the Effectors Ferulic Acid, Vanillin, and Vanillic Acid

**Figure 3:**
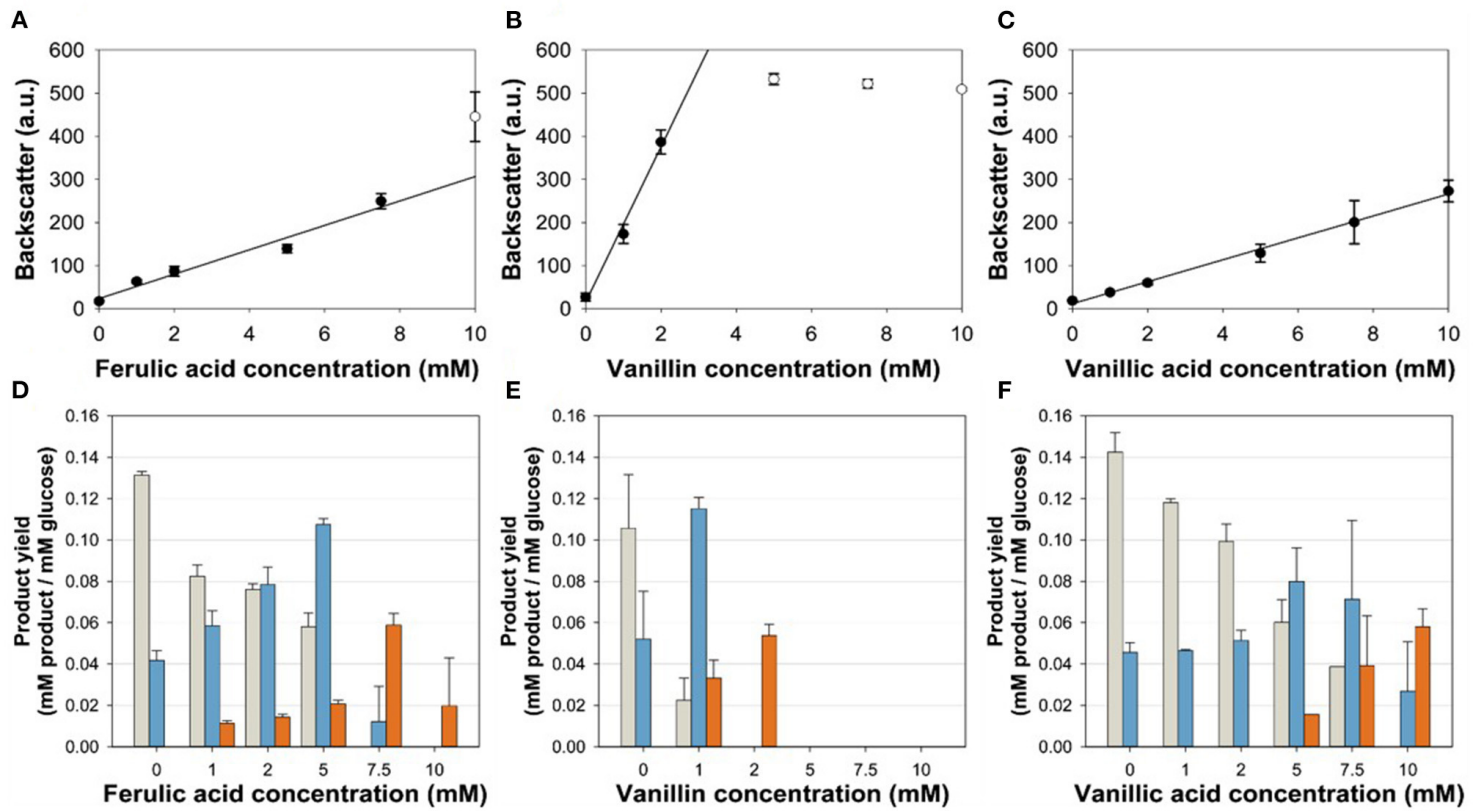
**(A,C)** Correlation of the final backscatter against the effector molecule concentration of *C. glutamicum* ΔP_*aceE*_::*vanR*-P_vanABK_* in microliter scale cultivations with CGXII minimal medium and 20 g glucose L^−1^ with different concentrations of **(A)** ferulic acid (*m* = 28 backscatter units/mM; *R*^2^ = 0.966), **(B)** vanillin (*m* = 180 backscatter units/mM; *R*^2^ = 0.989), and **(C)** vanillic acid (*m* = 25 backscatter units/mM; *R*^2^ = 0.996). Black line represents linear regression of black dots, while empty dots are excluded from this regression. **(D–F)** Product yields of the aforementioned cultivations for pyruvate (gray), alanine (blue), and valine (orange). Error bars represent standard deviation of at least three cultivations.

To test the suitability of the VanR/P_vanABK_^*^ regulatory system to dynamically control the gene expression in *C. glutamicum*, we constructed *C. glutamicum* ΔP_*aceE*_::*vanR*-P_vanABK_^*^ *via* exchange of the native *aceE* promotor by P_vanABK_^*^(Morabbi Heravi et al., 2015) and an additional copy of *vanR* (Figure 1). P_vanABK_^*^ carries a single point mutation in the −10 region (CAATAT → TAATAT) leading to a 73-fold induction compared with its native version (Morabbi Heravi et al., 2015) and we anticipated that the regulatory circuit drives *aceE* expression as long as the effector molecule is present in the medium. After utilization, growth should arrest due to the abolished *aceE* expression and, consequently, the loss of PDHC activity. *C. glutamicum* ΔP_*aceE*_::*vanR*-P_vanABK_^*^ was cultivated in a microliter scale (Biolector® I, M2PLabs, Baesweiler, Germany) with the CGXII minimal medium and 20 g glucose L^−1^ supplemented with 0–10 mM of the potential effector molecules FA, Van, and VA. Without an effector in the medium, *C. glutamicum* ΔP_*aceE*_::*vanR*-P_vanABK_^*^ showed negligible growth (Supplementary Figure 2). In the presence of the phenolic compounds, we observed a linear correlation between the introduced effector concentration and the finally reached biomass, but equimolar amounts led to different reads in the backscatter (Figures 3A–C; Supplementary Figure 2). While Van concentration >2 mM was already sufficient to reach final biomass comparable to the wild type (Supplementary Figures 1, 2), >7.5 mM FA was required and even 10 mM VA was not enough. No growth occurred for this mutant strain if PCA was added as effector molecule in the same concentration range (data not shown). The cell cultures were harvested at the end of the cultivation and the supernatants were analyzed by HPLC to determine the amounts of glucose and the three metabolites pyruvate, alanine, and valine. The product yields for all three metabolites showed a strong dependence on the applied effector molecule concentration. With increasing effector concentrations, the product spectrum shifted from pyruvate over alanine to valine, and at about half-maximal biomass concentration, highest cumulated product yields were observed (Figures 3D–F).

### Kinetic Analysis of *C. glutamicum* ΔP*_*aceE*_*::*vanR*-P_vanABK_^*^ and the Wild Type

**Figure 4:**
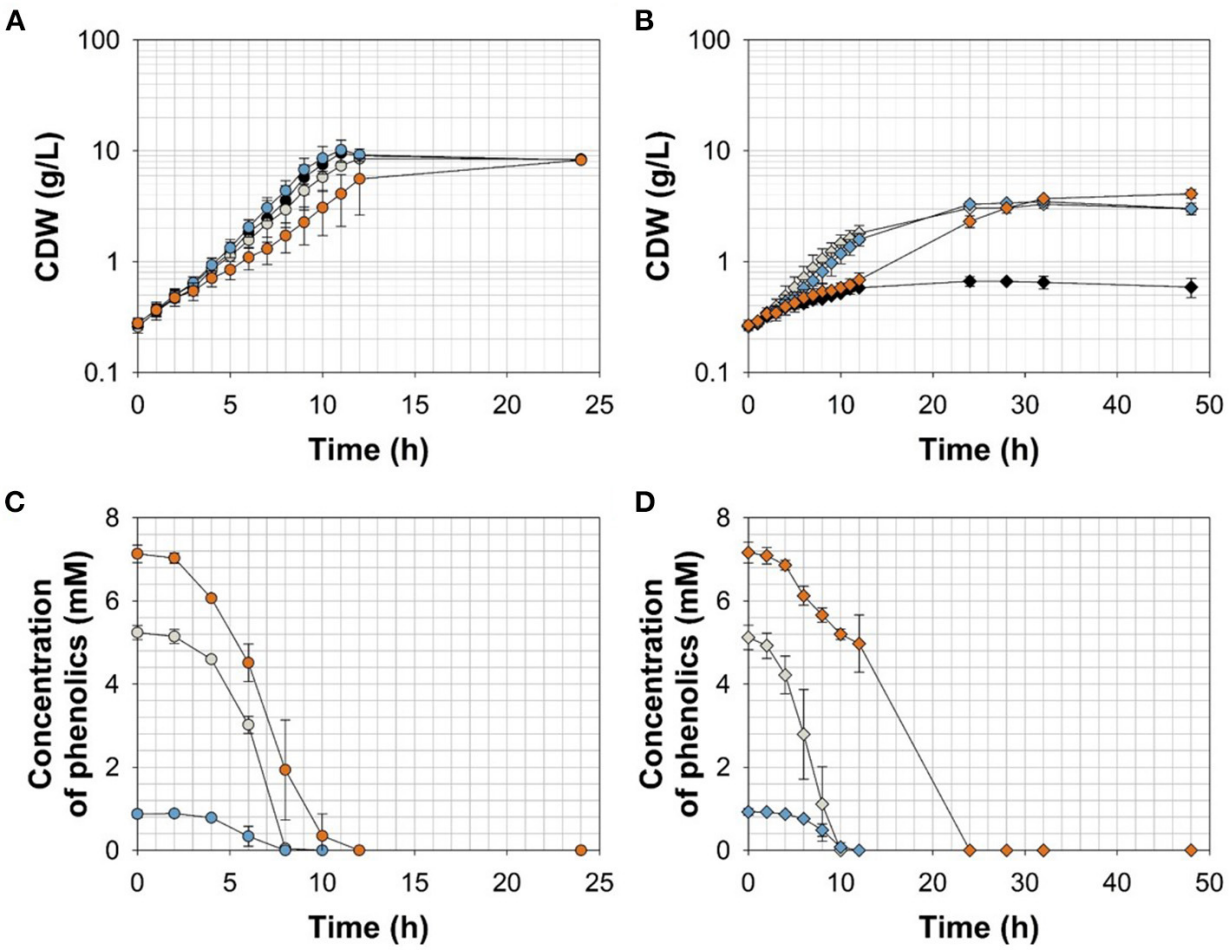
**(A,B)** Growth and **(C,D)** effector molecule consumption of *C. glutamicum* wild type (circles) and *C. glutamicum* ΔP_*aceE*_::*vanR*-P_vanABK_* (diamonds) in shaking flasks with CGXII minimal medium and 20 g glucose L^−1^ without (black) and with ferulic acid (gray), vanillin (blue), or vanillic acid (orange). Error bars represent standard deviation of cultivations of at least three biological replicates.

To further resolve the kinetics of growth and product formation in dependence of the effector molecules, we cultivated *C. glutamicum* ΔP_*aceE*_::*vanR*-P_vanABK_^*^ and the wild type in CGXII minimal medium with 20 g glucose L^−1^ at effector concentrations, which yielded around half of the maximal biomass concentration in comparison with the outgrown wild type. Thus, 5 mM FA, 1 mM Van, and 7.5 mM VA were applied (Figure 4; Table 3). The *C. glutamicum* wild type showed a growth rate of 0.35, 0.31, 0.39, and 0.23 h^−1^ in the medium without and with FA, Van, and VA (Figure 4A; Table 3), which is in accordance with the microscale cultivations (Figure 2). Furthermore, in the exponential growth phase, all the three phenolic compounds were consumed completely and in parallel to glucose (Figure 4C; Supplementary Figure 3A), which led to increased glucose-specific biomass yields correlating to the amount of additional carbon source provided by the phenolic compounds (Table 3). *C. glutamicum* did not secrete pyruvate, alanine, or valine into the culture broth. Interestingly, we observed the temporary accumulation of traces of VA in cultivations with FA (up to 0.1 mM VA) and Van (up to 0.03 mM VA; data not shown). *C. glutamicum* ΔP_*aceE*_::*vanR*-P_vanABK_^*^ showed 40–60% decreased growth rates compared with the wild type in the presence of the respective phenolic compounds and negligible growth without an effector (Figure 4B; Table 3). Furthermore, applying the correlation from the microscale cultivation (Figures 3A–C) allowed to adjust the final biomass concentration and the cells grew only until the effector molecule was fully depleted (Figures 4B,D). Although growth ceased, the cells remained metabolically active and started to secrete pyruvate, alanine, and valine (Supplementary Figures 3B–D; Table 3) and the reached product yields (Table 3) are in good accordance with the microliter scale experiments (Figures 3D–F). Also, the temporary accumulation of traces of VA, as described for the wild type, could be observed if the strain *C. glutamicum* ΔP_*aceE*_::*vanR*-P_vanABK_^*^ was cultivated with FA (up to 0.5 mM VA) and Van (up to 0.02 mM VA; data not shown).

**Table 3:** Growth rates, biomass yields, and product yields of *C. glutamicum* wild type, *C. glutamicum* ΔP_*aceE*_::*vanR*-P_vanABK_*, and *C. glutamicum* ΔP_*aceE*_::*vanR*-P_vanABK_* (pJC4-*ilvBNCE*) in shaking flasks with CGXII minimal medium and 20 g glucose L^−1^ without and with ferulic acid, vanillin, or vanillic acid. Standard deviation was determined *via* cultivations of at least three biological replicates.

**Concentration of effector molecule (mM)**	**Growth rate μ (h^**−1**^)**	**Biomass yield (g_**CDW**_/g_**glucose**_)**	**Pyruvate yield (mol/mol_**glucose**_)**	**Alanine yield (mol/mol_**glucose**_)**	**Valine yield (mol/mol_**glucose**_)**
***C. glutamicum*** **wild type**					
–	0.35 ± 0.01	0.47 ± 0.01	b.d.	b.d.	b.d.
FA (5.2 ± 0.2)	0.31 ± 0.03	0.52 ± 0.02	b.d.	b.d.	b.d.
Van (0.9 ± 0.1)	0.39 ± 0.01	0.50 ± 0.04	b.d.	b.d.	b.d.
VA (7.1 ± 0.2)	0.23 ± 0.04	0.58 ± 0.03	b.d.	b.d.	b.d.
***C. glutamicum*** **ΔP** *_***aceE***_* **::** ***vanR*** **-P** *_***vanABK***_* *					
–	n.d.	n.d.	0.18 ± 0.02	0.07 ± 0.01	b.d.
FA (5.1 ± 0.3)	0.19 ± 0.03	0.58 ± 0.09	0.08 ± 0.05	0.11 ± 0.02	0.01 ± 0.01
Van (0.9 ± 0.1)	0.16 ± 0.01	0.40 ± 0.01	0.06 ± 0.02	0.10 ± 0.01	0.02 ± 0.01
VA (7.2 ± 0.2)	0.10 ± 0.02	0.34 ± 0.03	0.07 ± 0.06	0.09 ± 0.01	0.02 ± 0.01
***C. glutamicum*** **ΔP** *_***aceE***_* **::** ***vanR*** **-P** *_***vanABK***_* * **(pJC4-** ***ilvBNCE*** **)**					
–	n.d.	n.d.	0.10 ± 0.05	0.04 ± 0.01	0.27 ± 0.03
FA (4.9 ± 0.1)	0.17 ± 0.01	0.52 ± 0.01	b.d.	0.02 ± 0.01	0.36 ± 0.02
Van (1.0 ± 0.1)	0.14 ± 0.02	0.34 ± 0.03	0.02 ± 0.03	0.03 ± 0.01	0.38 ± 0.01
VA (7.3 ± 0.1)	0.08 ± 0.01	0.38 ± 0.06	0.03 ± 0.05	0.03 ± 0.01	0.35 ± 0.04

*b.d., below detection limit; n.d., not determined*.

### Dynamically Induced Valine Production With *C. glutamicum* ΔP*_*aceE*_*::*vanR*-P_vanABK_^*^ Overexpressing *ilvBNCE*

**Figure 5:**
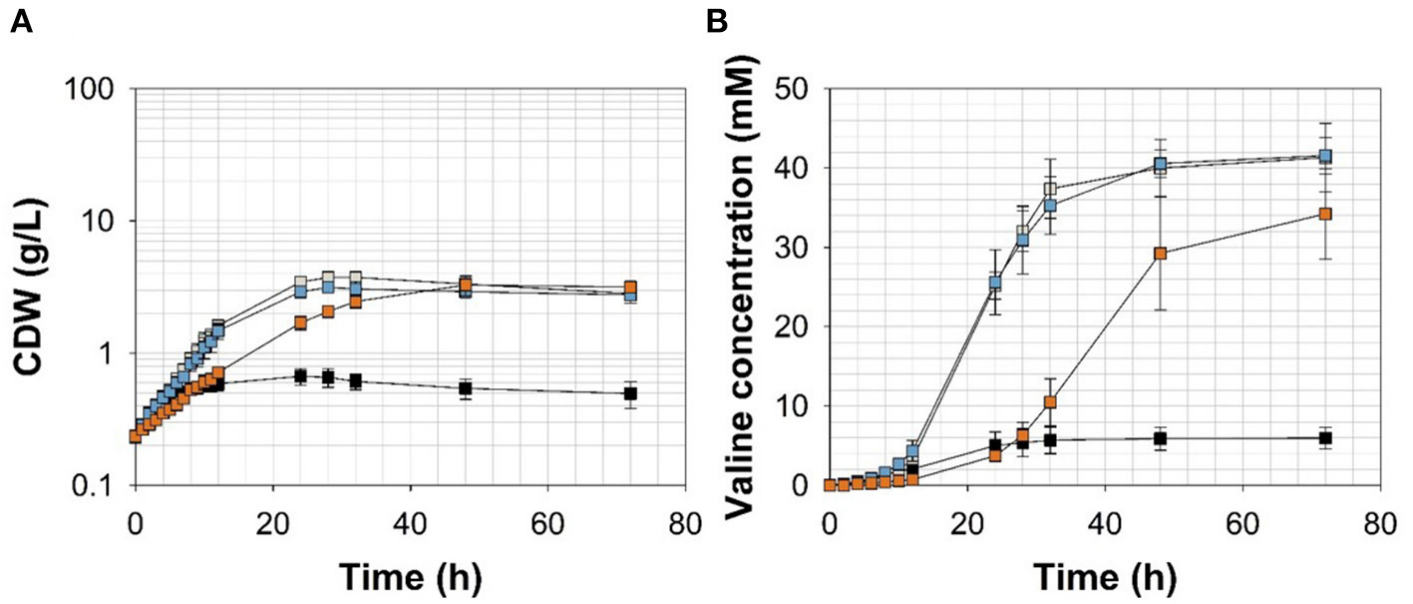
**(A)** Growth and **(B)** valine accumulation in the supernatant of *C. glutamicum*ΔP_*aceE*_::*vanR*-P_vanABK_*(pJC4-*ilvBNCE*) in shaking flasks with CGXII minimal medium (with 20 g ammonium sulfate L^−1^) and 20 g glucose L^−1^ without (black) and with ferulic acid (gray), vanillin (blue), or vanillic acid (orange) (for detailed concentration of effector molecules see Supplementary Figure 5; Table 3). Error bars represent standard deviation of cultivations of at least three biological replicates.

To channel the carbon flux more efficiently from pyruvate toward valine, we transformed *C. glutamicum* ΔP_*aceE*_::*vanR*-P_vanABK_^*^ with the plasmid pJC4-*ilvBNCE* (Radmacher et al., 2002) to overexpress the valine biosynthesis genes *ilvBNCE* encoding acetohydroxyacid synthase (*ilvBN*), isomeroreductase (*ilvC*), and transaminase B (*ilvE*). *C. glutamicum* ΔP_*aceE*_::*vanR*-P_vanABK_^*^ (pJC4-*ilvBNCE*) and the control strain *C. glutamicum* ΔP_*aceE*_::*vanR*-P_vanABK_^*^ (pJC4) were cultivated in shaking flasks with CGXII minimal medium and 20 g glucose L^−1^ supplemented with 5 mM FA, 1 mM Van, and 7.5 mM VA (Figure 5; Table 3). Under the conditions tested, *C. glutamicum* ΔP_*aceE*_::*vanR*-P_vanABK_^*^ and its plasmid carrying derivatives showed very similar growth and consumption of glucose and the phenolic compounds, and all the strains induced production after consumption of the respective effector molecule (Figures 5A, 4B; Supplementary Figures 5, 6; Table 3). *C. glutamicum* ΔP_*aceE*_::*vanR*-P_vanABK_^*^ and the derivative with the empty vector secreted similar amounts of pyruvate, alanine, and valine into the medium (Supplementary Figures 3B–D, 6C,E). However, overexpression of the *ilvBNCE* genes in *C. glutamicum* ΔP_*aceE*_::*vanR*-P_vanABK_^*^ shifted the product spectrum toward valine. In the medium containing FA, Van, or VA *C. glutamicum* ΔP_*aceE*_::*vanR*-P_vanABK_^*^ (pJC4-*ilvBNCE*) produced 40 ± 3.6, 40.6 ± 1.7, and 29.3 ± 7.1 mM valine after 48 h with a product yield of 0.36 ± 0.02, 0.38 ± 0.01, and 0.35 ± 0.04 mol valine per mol glucose (Figure 5B; Table 3). The concentration of pyruvate and alanine was, under all conditions, below 5 mM over the whole cultivation time (Supplementary Figures 4B,C).

## Discussion

In this study, the VanR/P_vanABK_^*^ regulatory system was utilized to dynamically control gene expression in *C. glutamicum*. We applied this circuit to switch off the *aceE* expression in a timed manner when the lignin-derived effectors FA, Van, and VA were depleted from the medium. Finally, we showed the feasibility of this approach to adjust biomass formation and to induce the production of valine.

The ability of *C. glutamicum* to tolerate higher concentrations of aromatics than other bacteria was already described (Shen et al., 2012; Morabbi Heravi et al., 2015; Becker and Wittmann, 2019) as well as to utilize the phenolic compounds FA, Van, and VA as sole carbon sources (Merkens et al., 2005; Brinkrolf et al., 2006; Shen et al., 2012; Kallscheuer et al., 2016). Here, we show that these molecules are also co-utilized with glucose (Figure 4C; Supplementary Figure 3A; Table 3), which is a promising feature of *C. glutamicum* with regard to the application of lignocellulosic hydrolysates as feedstock for industrial biotechnology. While minimum inhibitory concentrations (MIC) for the phenolic compounds tested in this study range for *E. coli* between 0.5 and 15 mM (Fitzgerald et al., 2004; Mourtzinos et al., 2009; Borges et al., 2013), *C. glutamicum* still shows, in the presence of up to 20 mM of each molecule, about 67–90% growth rate compared with reference conditions (Figure 2). Remarkably, low concentrations (up to 2 mM) of FA and Van significantly (*p*-value ≤ 0.01) increased the growth rate of *C. glutamicum* (Figure 2). It is known that diphenolic compounds, such as catechol or PCA, facilitate the growth of *C. glutamicum* (Liebl et al., 1989). Recently, it has been shown that this effect is based on the potential of diphenols, with two adjacent hydroxyl groups or a mix of amino and hydroxyl groups, to chelate iron and/or reduce Fe_3_^+^, thus improving the overall iron availability (Müller et al., 2020). If the positive effect of FA and Van is directly mediated by these molecules or if it is the effect of the degradation intermediate PCA (Brinkrolf et al., 2006; Shen et al., 2012; Kallscheuer et al., 2016) remains to be investigated. For VA, no positive effect on growth was observed at the applied concentrations (Figure 2). In general, VA has a much higher antimicrobial potential, especially in comparison with Van (Mourtzinos et al., 2009). Thus, it has to be tested if lower concentrations (< 1mM) of VA have a growth-promoting effect.

All the three phenolic compounds proved suitability to induce the VanR/P_vanABK_^*^ regulatory circuit, providing flexibility with regard to the intended application. However, since 2 mM VA already reduced the growth rate of the *C. glutamicum* wild type significantly (Figure 2; *p*-value ≤ 0.01), Van and FA are the preferred inducer molecules. VA, FA, and Van, compared with common inducers such as IPTG or rhamnose, are cheaper and abundant, since these molecules can be derived from lignocellulosic material. Therefore, VA, FA, and Van are, in principle, well-suited for industrial application if the main substrate of the process is not a lignocellulosic hydrolysate containing significant amounts of effector molecules. Future studies have to evaluate if the VanR/P_vanABK_^*^ regulatory circuit can be applied in large scale as well as in other hosts. As a prerequisite, the microbial system of choice should show considerable tolerance against the aromatic inducer molecules such as *Pseudomonads* (Becker and Wittmann, 2019). Moreover, if applied as synthetic off-switch, the organism should be able to metabolize the inducer molecules and its metabolism should not be under control of catabolite repression, which might result in inducer exclusion.

The final biomass of *C. glutamicum* ΔP_*aceE*_::*vanR*-P_vanABK_^*^ correlated directly with the applied amount of effector molecule. FA and VA showed almost identical slopes of the linear regression, while Van yielded an about 7-fold higher slope, i.e., seven times less Van has to be added to the culture in order to achieve the same biomass as with VA or FA (Figures 3A–C). The reason for this effect remains unclear, especially since in cultivations with FA and Van small amounts of the VanR effector VA were secreted into the medium, indicating a limitation in the conversion of VA to PCA catalyzed by vanillate demethylase VanAB. While biochemical data for VanAB are not available yet specifically for *C. glutamicum*, it is known that this enzyme complex shares significant similarity to VanABs of other microorganisms such as *Pseudomonas* species (Merkens et al., 2005; Brinkrolf et al., 2006), where it is described to convert VA to PCA, demanding NAD(P)H_2_ plus molecular oxygen and releasing NAD(P), water, and formaldehyde, which has to be further detoxified (Priefert et al., 1997; Hibi et al., 2005). This energy- and oxygen-demanding reaction, along with the release of formaldehyde, is a good indicator for the strict regulation of VanABK in *C. glutamicum* (Merkens et al., 2005; Brinkrolf et al., 2006; Morabbi Heravi et al., 2015).

The PDHC of *C. glutamicum* is a promising target for metabolic engineering (Eikmanns and Blombach, 2014), and it has been shown that the PDHC-deficient strain *C. glutamicum* Δ*aceE* is unable to grow on glucose unless supplemented with acetate (Schreiner et al., 2005). This feature has been utilized to adjust biomass formation over the provided amount of acetate. After its depletion, the growth of *C. glutamicum*Δ*aceE* stopped, but the cells remained metabolically active and started to produce pyruvate, alanine, and valine from glucose (Blombach et al., 2007). More recently, Wiechert et al. (2020) designed an inducible metabolic toggle switch, which efficiently controls the *aceE* expression by the effector molecule gluconate. However, compared with acetate as well as gluconate as inducers, at least 50 times lower molar amounts of Van have to be introduced into the process to achieve similar biomass concentrations (Blombach et al., 2007; Wiechert et al., 2020).

Compared with *C. glutamicum* P_*gntK*_-*aceE* (Wiechert et al., 2020), *C. glutamicum* ΔP_*aceE*_::*vanR*-P_vanABK_^*^ showed an around 50% slower growth rate with Van and FA, indicating further potential to optimize the VanR/P_vanABK_^*^ system. Data relating the promotor strength of P_*aceE*_, P_vanABK_^*^, and P_gntK_ under comparable conditions are not available. However, while the ribosomal binding site “AGGAG” was utilized in the promoter regions of P_gntK_ and P_vanABK_^*^ (Wiechert et al., 2020), the spacer regions are quite different, potentially impacting gene expression (Schneider et al., 2012).

In microliter cultivations, the product pattern of *C. glutamicum* ΔP_*aceE*_::*vanR*-P_vanABK_^*^ (Figures 3D–F) shifted from pyruvate over alanine to valine with increasing effector and final biomass concentrations. This effect was observed for all three phenolic compounds, indicating biomass-dependent metabolic changes, which promote valine synthesis. However, to channel the carbon flux toward valine, plasmid-based overexpression of the valine biosynthesis genes *ilvBNCE* was more efficient. *C. glutamicum* ΔP_*aceE*_::*vanR*-P_vanABK_^*^ (pJC4-*ilvBNCE*) showed product yields of about 0.36 mol valine per mol glucose, which are about 6-fold higher compared with the yields of *C. glutamicum* ΔP_*aceE*_::*vanR*-P_vanABK_^*^ at optimal effector concentrations (Figures 3D–F; Table 3). Although the glucose-based product yield of *C. glutamicum* ΔP_*aceE*_::*vanR*-P_vanABK_^*^ (pJC4-*ilvBNCE*) is considerably lower in comparison with 0.47 mol valine per mol glucose of *C. glutamicum*Δ*aceE* (pJC4-*ilvBNCE*) and 0.52 mol valine per mol glucose of *C. glutamicum* P_*gntK*_-*aceE* (pJC4-P_*ilvB*_-*ilvBNC*-P_*ilvE*_-*ilvE*), the overall yield (including the amount of effector) of 0.36 C-mol per C-mol of *C. glutamicum* ΔP_*aceE*_::*vanR*-P_vanABK_^*^ (pJC4-*ilvBNCE*) with Van is at least identical to the latter two strains with values of 0.32 and 0.36 C-mol per C-mol, respectively (Blombach et al., 2007; Wiechert et al., 2020).

Concluding, this study shows the suitability of the VanR/P_vanABK_^*^ system as a tool to control gene expression in *C. glutamicum*. The regulatory circuit can be toggled by the cheap and abundant lignin-derived phenolic compounds FA, Van, and VA. Since these three molecules are consumed in parallel to the main substrate glucose, the regulatory circuit represents a valuable genetic control element to adjust gene expression on demand.

## Data Availability Statement

The original contributions presented in the study are included in the article/Supplementary Material. Further inquiries can be directed to the corresponding author/s.

## Author Contributions

DS and BB conceived and supervised the study and designed the experiments. DS performed the experiments and analyzed the data. DS, JA, and BB wrote the manuscript. All authors contributed to the article and approved the submitted version.

## Conflict of Interest

The authors declare that the research was conducted in the absence of any commercial or financial relationships that could be construed as a potential conflict of interest.

## Publisher's Note

All claims expressed in this article are solely those of the authors and do not necessarily represent those of their affiliated organizations, or those of the publisher, the editors and the reviewers. Any product that may be evaluated in this article, or claim that may be made by its manufacturer, is not guaranteed or endorsed by the publisher.

## References

[B1] Abe S. Takayama K.-I. Kinoshita S. (1967). Taxonomical studies on glutamic acid-producing bacteria. J. Gen. Appl. Microbiol. 13, 279–301. 10.2323/jgam.13.279

[B2] Agilent Technologies (2020). Agilent Biocolumns: Amino Acid Analysis. “How-To” Guide. Available online at: https://www.agilent.com/cs/library/brochures/5991-7694EN_AdvanceBio%20AAA_How-To%20Guide_LR.pdf (accessed March 29, 2021).

[B3] Ball S. Bullock S. Lloyd L. Mapp K. (2011). Agilent Hi-Plex Columns Applications Compendium. Available online at: https://www.agilent.com/cs/library/applications/5990-8801EN%20Hi-Plex%20Compendium.pdf (accessed March 29, 2021).

[B4] Baumschlager A. Rullan M. Khammash M. (2020). Exploiting natural chemical photosensitivity of anhydrotetracycline and tetracycline for dynamic and setpoint chemo-optogenetic control. Nat. Comm. 11:3834. 10.1038/s41467-020-17677-5 32737309 PMC7395757

[B5] Becker J. Rohles C. M. Wittmann C. (2018). Metabolically engineered *Corynebacterium glutamicum* for bio-based production of chemicals, fuels, materials, and healthcare products. Metab. Eng. 50, 122–141. 10.1016/j.ymben.2018.07.008 30031852

[B6] Becker J. Wittmann C. (2019). A field of dreams: Lignin valorization into chemicals, materials, fuels, and health-care products. Biotechnol. Adv. 37:107360. 10.1016/j.biotechadv.2019.02.016 30959173

[B7] Blombach B. Eikmanns B. J. (2011). Current knowledge on isobutanol production with *Escherichia coli, Bacillus subtilis* and *Corynebacterium glutamicum*. Bioeng. Bugs. 2, 346–350. 10.4161/bbug.2.6.17845 22008938 PMC3242789

[B8] Blombach B. Riester T. Wieschalka S. Ziert C. Youn J.-W. Wendisch V. F.. (2011). *Corynebacterium glutamicum* tailored for efficient isobutanol production. Appl. Environ. Microb. 77, 3300–3310. 10.1128/AEM.02972-10 21441331 PMC3126470

[B9] Blombach B. Schreiner M. E. Holátko J. Bartek T. Oldiges M. Eikmanns B. J. (2007). L-valine production with pyruvate dehydrogenase complex-deficient *Corynebacterium glutamicum*. Appl. Environ. Microb. 73, 2079–2084. 10.1128/AEM.02826-06 17293513 PMC1855657

[B10] Borges A. Ferreira C. Saavedra M. J. Simões M. (2013). Antibacterial activity and mode of action of ferulic and gallic acids against pathogenic bacteria. Microb. Drug. Resist. 19, 256–265. 10.1089/mdr.2012.0244 23480526

[B11] Brautaset T. Lale R. Valla S. (2009). Positively regulated bacterial expression systems. Microb. Biotechnol. 2, 15–30. 10.1111/j.1751-7915.2008.00048.x 21261879 PMC3815419

[B12] Brinkrolf K. Brune I. Tauch A. (2006). Transcriptional regulation of catabolic pathways for aromatic compounds in *Corynebacterium glutamicum*. Genet. Mol. Res 5, 773–789. 17183485

[B13] Buchholz J. Graf M. Blombach B. Takors R. (2014). Improving the carbon balance of fermentations by total carbon analyses. Biochem. Eng. J. 90, 162–169. 10.1016/j.bej.2014.06.007

[B14] Burkovski A.(ed.) (2008). Corynebacteria: Genomics and Molecular Biology. Wymondham: Caister Academic.

[B15] Cardoso V. M. Campani G. Santos M. P. Silva G. G. Pires M. C. Gonçalves V. M.. (2020). Cost analysis based on bioreactor cultivation conditions: Production of a soluble recombinant protein using *Escherichia coli* BL21(DE3). Biotechnol. Rep. 26:e00441. 10.1016/j.btre.2020.e00441 32140446 PMC7049567

[B16] Chaudhry M. T. Huang Y. Shen X.-H. Poetsch A. Jiang C.-Y. Liu S.-J. (2007). Genome-wide investigation of aromatic acid transporters in *Corynebacterium glutamicum*. Microbiology 153, 857–865. 10.1099/mic.0.2006/002501-0 17322206

[B17] Cordes C. Möckel B. Eggeling L. Sahm H. (1992). Cloning, organization and functional analysis of *ilvA, ilvB* and *ilvC* genes from *Corynebacterium glutamicum*. Gene 112, 113–116. 10.1016/0378-1119(92)90311-C 1551588

[B18] Ding W. Si M. Zhang W. Zhang Y. Chen C. Zhang L.. (2015). Functional characterization of a vanillin dehydrogenase in *Corynebacterium glutamicum*. Sci. Rep. 5:8044. 10.1038/srep08044 25622822 PMC4306973

[B19] Eggeling L. Bott M.(eds) (2005). Handbook of Corynebacterium glutamicum. Boca Raton, FL: CRC Press.

[B20] Eikmanns B. J. Blombach B. (2014). The pyruvate dehydrogenase complex of *Corynebacterium glutamicum*: an attractive target for metabolic engineering. J. Biotechnol. 192 (Pt B0), 339–345. 10.1016/j.jbiotec.2013.12.019 24486441

[B21] Ferreira R. d. G Azzoni A. R. Freitas S. (2018). Techno-economic analysis of the industrial production of a low-cost enzyme using *E. coli*: the case of recombinant β-glucosidase. Biotechnol. Biofuels. 11:81. 10.1186/s13068-018-1077-0 29610578 PMC5875018

[B22] Fitzgerald D. J. Stratford M. Gasson M. J. Ueckert J. Bos A. Narbad A. (2004). Mode of antimicrobial action of vanillin against *Escherichia coli, Lactobacillus plantarum* and *Listeria innocua*. J. Appl. Microbiol. 97, 104–113. 10.1111/j.1365-2672.2004.02275.x 15186447

[B23] Gauttam R. Desiderato C. Jung L. Shah A. Eikmanns B. J. (2019). A step forward: compatible and dual-inducible expression vectors for gene co-expression in *Corynebacterium glutamicum*. Plasmid 101, 20–27. 10.1016/j.plasmid.2018.12.004 30594649

[B24] Gibson D. G. (2011). Enzymatic assembly of overlapping DNA fragments. Method. Enzymol. 498, 349–361. 10.1016/B978-0-12-385120-8.00015-2 21601685 PMC7149801

[B25] Glasscock C. J. Biggs B. W. Lazar J. T. Arnold J. H. Burdette L. A. Valdes A.. (2021). Dynamic control of gene expression with riboregulated switchable feedback promoters. ACS. Synth. Biol. 10, 1199–1213. 10.1021/acssynbio.1c00015 33834762 PMC8141045

[B26] Goldbeck O. Seibold G. M. (2018). Construction of pOGOduet - An inducible, bicistronic vector for synthesis of recombinant proteins in *Corynebacterium glutamicum*. Plasmid 95, 11–15. 10.1016/j.plasmid.2018.01.001 29331350

[B27] Green M. R. Sambrook J. (2012). MOLECULAR cloning: A Laboratory Manual. Cold Spring Harbor, N.Y: Cold Spring Harbor Laboratory Press.

[B28] Hanahan D. (1983). Studies on transformation of *Escherichia coli* with plasmids. J. Mol. Biol. 166, 557–580. 10.1016/S0022-2836(83)80284-8 6345791

[B29] Hasegawa S. Jojima T. Suda M. Inui M. (2020). Isobutanol production in *Corynebacterium glutamicum*: suppressed succinate by-production by *pckA* inactivation and enhanced productivity via the Entner-Doudoroff pathway. Metab. Eng. 59, 24–35. 10.1016/j.ymben.2020.01.004 31926306

[B30] Hibi M. Sonoki T. Mori H. (2005). Functional coupling between vanillate-O-demethylase and formaldehyde detoxification pathway. FEMS Microbiol. Lett. 253, 237–242. 10.1016/j.femsle.2005.09.036 16242864

[B31] Ikeda M. Nakagawa S. (2003). The *Corynebacterium glutamicum* genome: features and impacts on biotechnological processes. Appl. Microbiol. Biot. 62, 99–109. 10.1007/s00253-003-1328-1 12743753

[B32] Inui M. Kawaguchi H. Murakami S. Vertès A. A. Yukawa H. (2004). Metabolic engineering of *Corynebacterium glutamicum* for fuel ethanol production under oxygen-deprivation conditions. J. Mol. Microb. Biotech. 8, 243–254. 10.1159/000086705 16179801

[B33] Inui M. Toyoda K. (2020). Corynebacterium glutamicum: Biology and Biotechnology. Cham: Springer International Publishing.

[B34] Jayaraman P. Yeoh J. W. Zhang J. Poh C. L. (2018). Programming the dynamic control of bacterial gene expression with a chimeric ligand- and light-lased promoter system. ACS Synth. Biol. 7, 2627–2639. 10.1021/acssynbio.8b00280 30359530

[B35] Kalinowski J. Bathe B. Bartels D. Bischoff N. Bott M. Burkovski A.. (2003). The complete *Corynebacterium glutamicum* ATCC 13032 genome sequence and its impact on the production of L-aspartate-derived amino acids and vitamins. J. Biotechnol. 104, 5–25. 10.1016/S0168-1656(03)00154-8 12948626

[B36] Kallscheuer N. Vogt M. Kappelmann J. Krumbach K. Noack S. Bott M.. (2016). Identification of the *phd* gene cluster responsible for phenylpropanoid utilization in *Corynebacterium glutamicum*. Appl. Microbiol. Biot. 100, 1871–1881. 10.1007/s00253-015-7165-1 26610800

[B37] Kind S. Wittmann C. (2011). Bio-based production of the platform chemical 1,5-diaminopentane. Appl. Microbiol. Biot. 91, 1287–1296. 10.1007/s00253-011-3457-2 21761208

[B38] Lange J. Müller F. Takors R. Blombach B. (2018). Harnessing novel chromosomal integration loci to utilize an organosolv-derived hemicellulose fraction for isobutanol production with engineered *Corynebacterium glutamicum*. Microb. Biotechnol. 11, 257–263. 10.1111/1751-7915.12879 29115043 PMC5743825

[B39] Lee S. K. Keasling J. D. (2005). A propionate-inducible expression system for enteric bacteria. Appl. Environ. Microb. 71, 6856–6862. 10.1128/AEM.71.11.6856-6862.2005 16269719 PMC1287719

[B40] Liebl W. Klamer R. Schleifer K.-H. (1989). Requirement of chelating compounds for the growth of *Corynebacterium glutamicum* in synthetic media. Appl. Microbiol. Biot. 32, 205–210. 10.1007/BF00165889

[B41] Menkel E. Thierbach G. Eggeling L. Sahm H. (1989). Influence of increased aspartate availability on lysine formation by a recombinant strain of *Corynebacterium glutamicum* and utilization of fumarate. Appl. Environ. Microb. 55, 684–688. 10.1128/aem.55.3.684-688.1989 2494939 PMC184180

[B42] Merkens H. Beckers G. Wirtz A. Burkovski A. (2005). Vanillate metabolism in *Corynebacterium glutamicum*. Curr. Microbiol. 51, 59–65. 10.1007/s00284-005-4531-8 15971090

[B43] Mollerup Andersen J. Batsberg Pedersen W. (1983). Analysis of plant phenolics by high-performance liquid chromatography. J. Chromatogr. A 259, 131–139. 10.1016/S0021-9673(01)87986-3

[B44] Morabbi Heravi K. Lange J. Watzlawick H. Kalinowski J. Altenbuchner J. (2015). Transcriptional regulation of the vanillate utilization genes (*vanABK* Operon) of *Corynebacterium glutamicum* by VanR, a PadR-like repressor. J. Bacteriol. 197, 959–972. 10.1128/JB.02431-14 25535273 PMC4325110

[B45] Mourtzinos I. Konteles S. Kalogeropoulos N. Karathanos V. T. (2009). Thermal oxidation of vanillin affects its antioxidant and antimicrobial properties. Food Chem. 114, 791–797. 10.1016/j.foodchem.2008.10.014

[B46] Müller F. Rapp J. Hacker A.-L. Feith A. Takors R. Blombach B. (2020). CO_2_/HCO_3_^-^ accelerates iron reduction through phenolic compounds. mBio. 11. 10.1128/mBio.00085-20 32156807 PMC7064749

[B47] Nishimura T. Vertès A. A. Shinoda Y. Inui M. Yukawa H. (2007). Anaerobic growth of *Corynebacterium glutamicum* using nitrate as a terminal electron acceptor. Appl. Microbiol. Biot. 75, 889–897. 10.1007/s00253-007-0879-y 17347820

[B48] Okai N. Masuda T. Takeshima Y. Tanaka K. Yoshida K.-I. Miyamoto M.. (2017). Biotransformation of ferulic acid to protocatechuic acid by *Corynebacterium glutamicum* ATCC 21420 engineered to express vanillate O-demethylase. AMB Exp. 7:130. 10.1186/s13568-017-0427-9 28641405 PMC5479773

[B49] Oldiges M. Eikmanns B. J. Blombach B. (2014). Application of metabolic engineering for the biotechnological production of L-valine. Appl. Microbiol. Biot. 98, 5859–5870. 10.1007/s00253-014-5782-8 24816722

[B50] Pfeifer-Sancar K. Mentz A. Rückert C. Kalinowski J. (2013). Comprehensive analysis of the *Corynebacterium glutamicum* transcriptome using an improved RNAseq technique. BMC Genom. 14:888. 10.1186/1471-2164-14-888 24341750 PMC3890552

[B51] Priefert H. Rabenhorst J. Steinbüchel A. (1997). Molecular characterization of genes of *Pseudomonas sp*. strain HR199 involved in bioconversion of vanillin to protocatechuate. J. Bacteriol. 179, 2595–2607. 10.1128/jb.179.8.2595-2607.1997 9098058 PMC179009

[B52] Radmacher E. Vaitsikova A. Burger U. Krumbach K. Sahm H. Eggeling L. (2002). Linking central metabolism with increased pathway flux: L-valine accumulation by *Corynebacterium glutamicum*. Appl. Environ. Microb. 68, 2246–2250. 10.1128/AEM.68.5.2246-2250.2002 11976094 PMC127577

[B53] Schäfer A. Tauch A. Jäger W. Kalinowski J. Thierbach G. Pühler A. (1994). Small mobilizable multi-purpose cloning vectors derived from the *Escherichia coli* plasmids pK18 and pK19: selection of defined deletions in the chromosome of *Corynebacterium glutamicum*. Gene 145, 69–73. 10.1016/0378-1119(94)90324-7 8045426

[B54] Schneider J. Eberhardt D. Wendisch V. F. (2012). Improving putrescine production by *Corynebacterium glutamicum* by fine-tuning ornithine transcarbamoylase activity using a plasmid addiction system. Appl. Microbiol. Biot. 95, 169–178. 10.1007/s00253-012-3956-9 22370950

[B55] Schreiner M. E. Fiur D. Holátko J. Pátek M. Eikmanns B. J. (2005). E1 enzyme of the pyruvate dehydrogenase complex in *Corynebacterium glutamicum*: molecular analysis of the gene and phylogenetic aspects. J. Bacteriol. 187, 6005–6018. 10.1128/JB.187.17.6005-6018.2005 16109942 PMC1196148

[B56] Schwentner A. Feith A. Münch E. Busche T. Rückert C. Kalinowski J.. (2018). Metabolic engineering to guide evolution - Creating a novel mode for L-valine production with *Corynebacterium glutamicum*. Metab. Eng. 47, 31–41. 10.1016/j.ymben.2018.02.015 29522826

[B57] Schwentner A. Feith A. Münch E. Stiefelmaier J. Lauer I. Favilli L.. (2019). Modular systems metabolic engineering enables balancing of relevant pathways for L-histidine production with *Corynebacterium glutamicum*. Biotechnol. Biofuels 12:65. 10.1186/s13068-019-1410-2 30962820 PMC6432763

[B58] Shen X.-H. Zhou N.-Y. Liu S.-J. (2012). Degradation and assimilation of aromatic compounds by *Corynebacterium glutamicum*: another potential for applications for this bacterium? Appl. Microbiol. Biot. 95, 77–89. 10.1007/s00253-012-4139-4 22588501

[B59] Siebert D. Wendisch V. F. (2015). Metabolic pathway engineering for production of 1,2-propanediol and 1-propanol by *Corynebacterium glutamicum*. Biotechnol. Biofuels. 8:91. 10.1186/s13068-015-0269-0 26110019 PMC4478622

[B60] Takeno S. Ohnishi J. Komatsu T. Masaki T. Sen K. Ikeda M. (2007). Anaerobic growth and potential for amino acid production by nitrate respiration in *Corynebacterium glutamicum*. Appl. Microbiol. Biot. 75, 1173–1182. 10.1007/s00253-007-0926-8 17380327

[B61] Tauch A. Kirchner O. Löffler B. Götker S. Pühler A. Kalinowski J. (2002). Efficient electrotransformation of *Corynebacterium diphtheriae* with a mini-replicon derived from the *Corynebacterium glutamicum* plasmid pGA1. Curr. Microbiol. 45, 362–367. 10.1007/s00284-002-3728-3 12232668

[B62] Terpe K. (2006). Overview of bacterial expression systems for heterologous protein production: from molecular and biochemical fundamentals to commercial systems. Appl. Microbiol. Biot. 72, 211–222. 10.1007/s00253-006-0465-8 16791589

[B63] van der Rest M. E. Lange C. Molenaar D. (1999). A heat shock following electroporation induces highly efficient transformation of *Corynebacterium glutamicum* with xenogeneic plasmid DNA. Appl. Microbiol. Biot. 52, 541–545. 10.1007/s002530051557 10570802

[B64] Wang Q. Zhang J. Al Makishah N. H. Sun X. Wen Z. Jiang Y.. (2021). Advances and perspectives for genome editing tools of *Corynebacterium glutamicum*. Front. Microbiol. 12:654058. 10.3389/fmicb.2021.654058 33897668 PMC8058222

[B65] Wendisch V. F. (2017). Microbial production of amino acid-related compounds, in Amino Acid Fermentation, eds Yokota A. Ikeda M. (Tokyo: Springer Japan), 255–269. 10.1007/10_2016_34 27872963

[B66] Wiechert J. Gätgens C. Wirtz A. Frunzke J. (2020). Inducible expression systems based on xenogeneic silencing and counter-silencing and design of a metabolic toggle switch. ACS Synth. Biol. 9, 2023–2038. 10.1021/acssynbio.0c00111 32649183 PMC7116053

[B67] Yamamoto S. Suda M. Niimi S. Inui M. Yukawa H. (2013). Strain optimization for efficient isobutanol production using *Corynebacterium glutamicum* under oxygen deprivation. Biotechnol. Bioeng. 110, 2938–2948. 10.1002/bit.24961 23737329

[B68] Yao J. He Y. Su N. Bharath S. R. Tao Y. Jin J.-M.. (2020). Developing a highly efficient hydroxytyrosol whole-cell catalyst by de-bottlenecking rate-limiting steps. Nat. Comm. 11:1515. 10.1038/s41467-020-14918-5 32251291 PMC7090077

[B69] Yukawa H. Inui M.(eds.) (2013). Corynebacterium glutamicum. Berlin, Heidelberg: Springer Berlin Heidelberg.

