# Supplementary material for: A Timed Off-Switch for Dynamic Control of Gene Expression in Corynebacterium Glutamicum
Source: Front Bioeng Biotechnol. 2021 Jul 29;9:704681. doi: 10.3389/fbioe.2021.704681 (PMC8358305; doi:10.3389/fbioe.2021.704681)
Supplement: Supplementary file 1 [file Data_Sheet_1.docx]

Supplementary Material

# Growth of *C. glutamicum* wild type on glucose in the absence and presence of the phenolic compounds ferulic acid, vanillin and vanillic acid


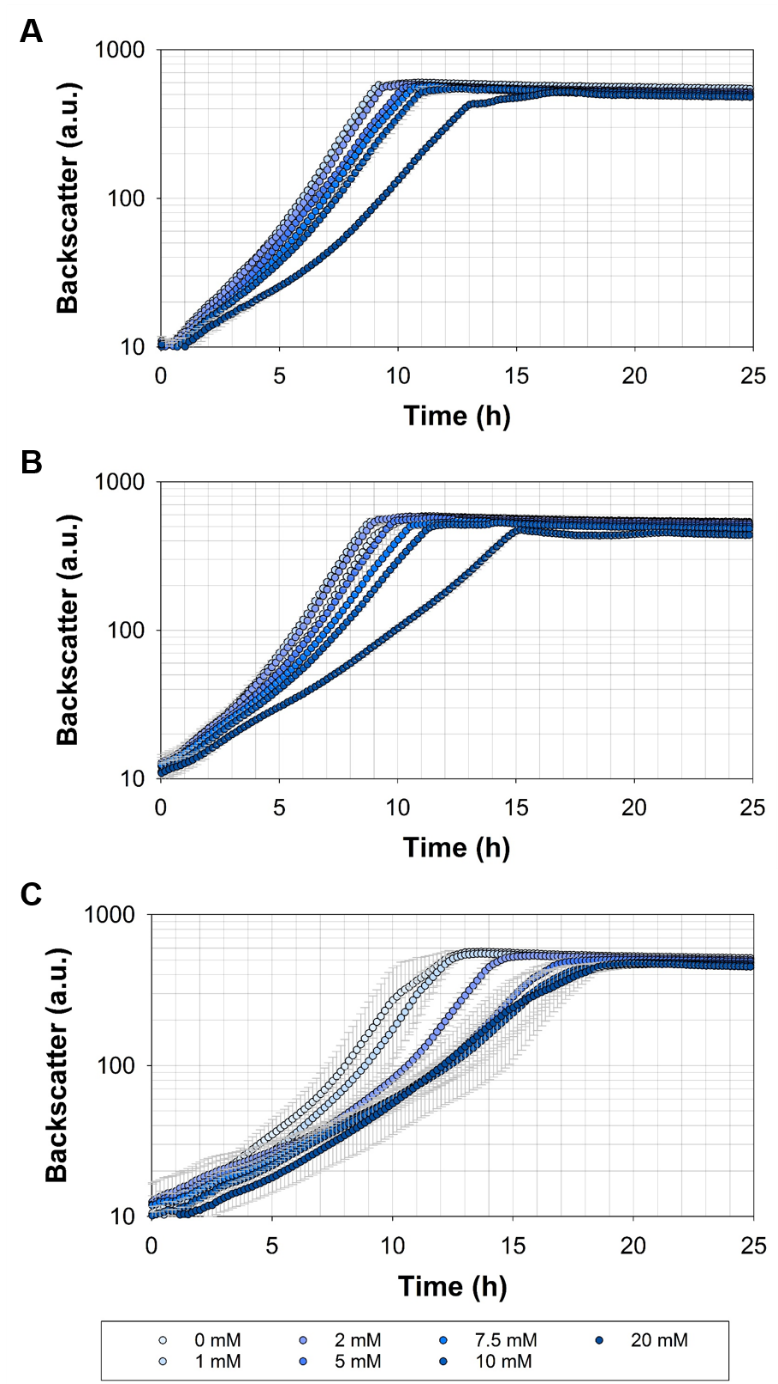


Figure S1: Growth of *C. glutamicum* wild type in microliter scale cultivations with CGXII minimal medium and 20 g glucose L^-1^ with different concentrations (shades of blue) of (A) ferulic acid, (B) vanillin and (C) vanillic acid. Error bars represent standard deviation of at least three cultivations.

# Growth of *C. glutamicum* ΔP*_aceE_*::*vanR*-P*_vanABK_** on glucose in the absence and presence of the phenolic compounds ferulic acid, vanillin and vanillic acid


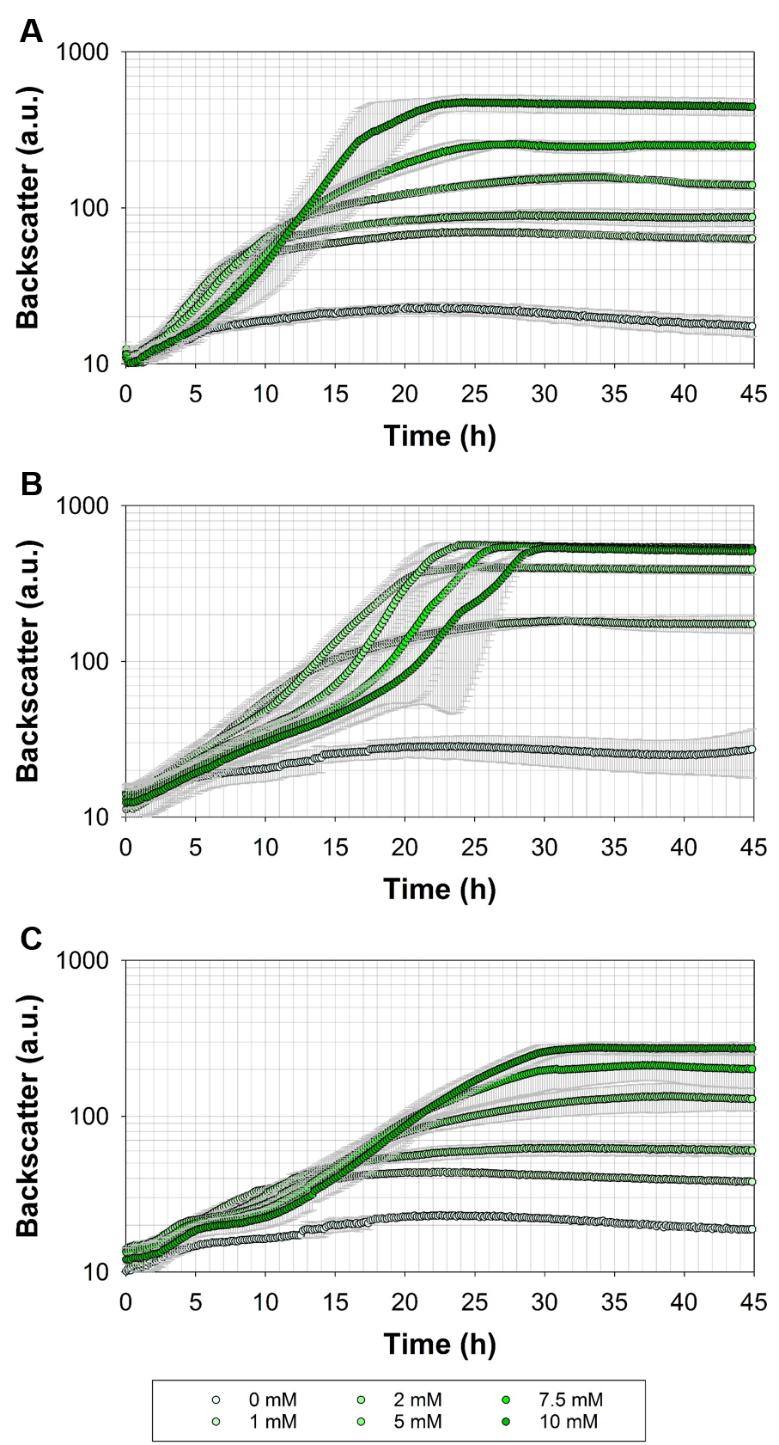


Figure S2: Growth of *C. glutamicum* ΔP*_aceE_*::*vanR*-P*_vanABK_** in microliter scale cultivations with CGXII minimal medium and 20 g glucose L^-1^ with different concentrations (shades of green) of (A) ferulic acid, (B) vanillin and (C) vanillic acid. Error bars represent standard deviation of at least three cultivations.

# Glucose consumption and product accumulation of *C. glutamicum* wild type and ΔP*_aceE_*::*vanR*-P*_vanABK_** in shaking flask cultivations supplemented with one specific concentration of ferulic acid, vanillin or vanillic acid


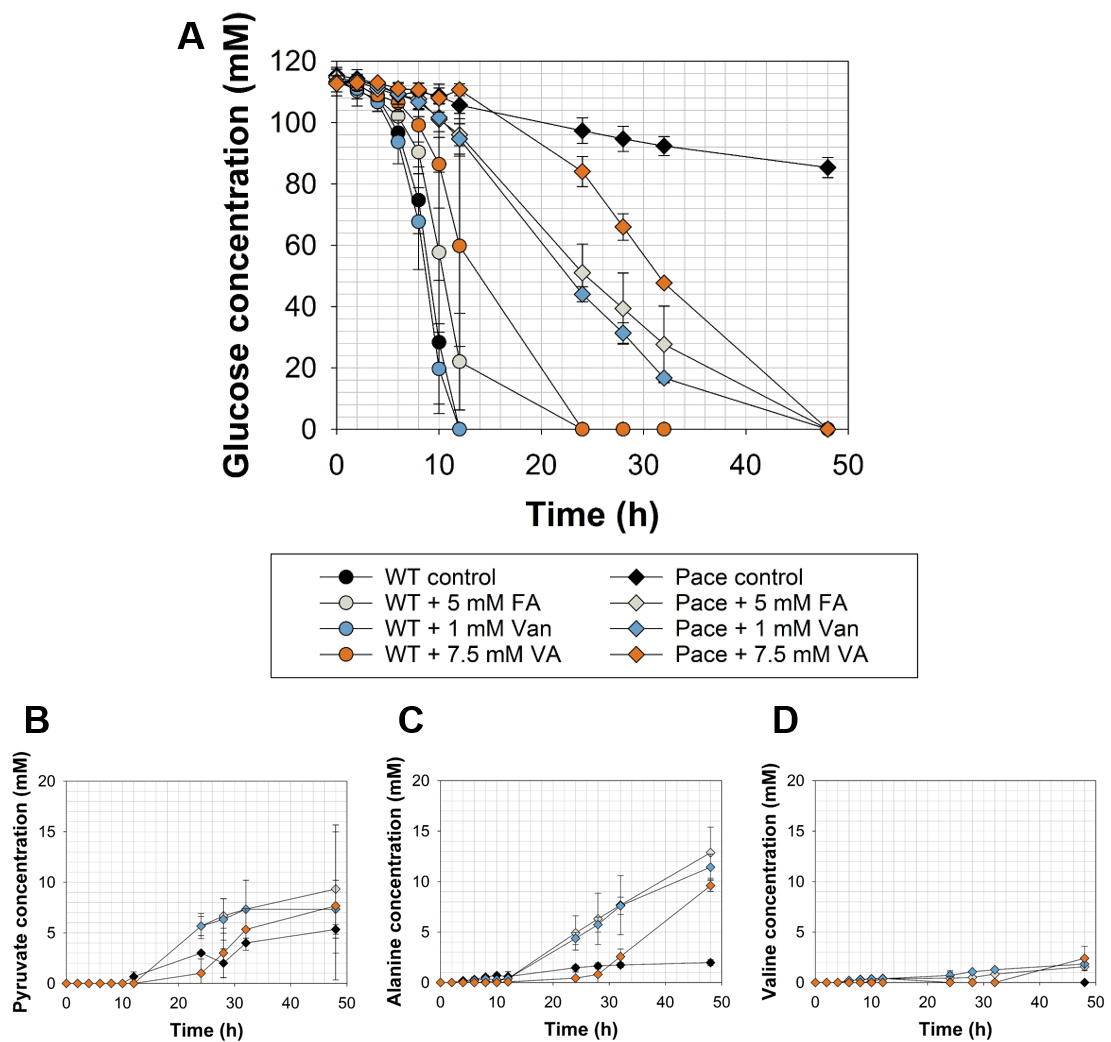


Figure S3: (A) Glucose consumption and (B – D) product accumulation of *C. glutamicum* wild type (circles; WT) and ΔP*_aceE_*::*vanR*-P*_vanABK_** (diamonds; Pace) in shaking flasks with CGXII minimal medium and 20 g glucose L^-1^ without (black) and with 5 mM ferulic acid (grey; FA), 1 mM vanillin (blue; Van) or 7.5 mM vanillic acid (orange; VA). Error bars represent standard deviation of cultivations of at least three biological replicates.

# Consumption of glucose and accumulation of pyruvate and alanine of *C. glutamicum* ΔP*_aceE_*::*vanR*-P*_vanABK_** (pJC4-*ilvBNCE*) in shaking flask cultivations supplemented with one specific concentration of ferulic acid, vanillin or vanillic acid


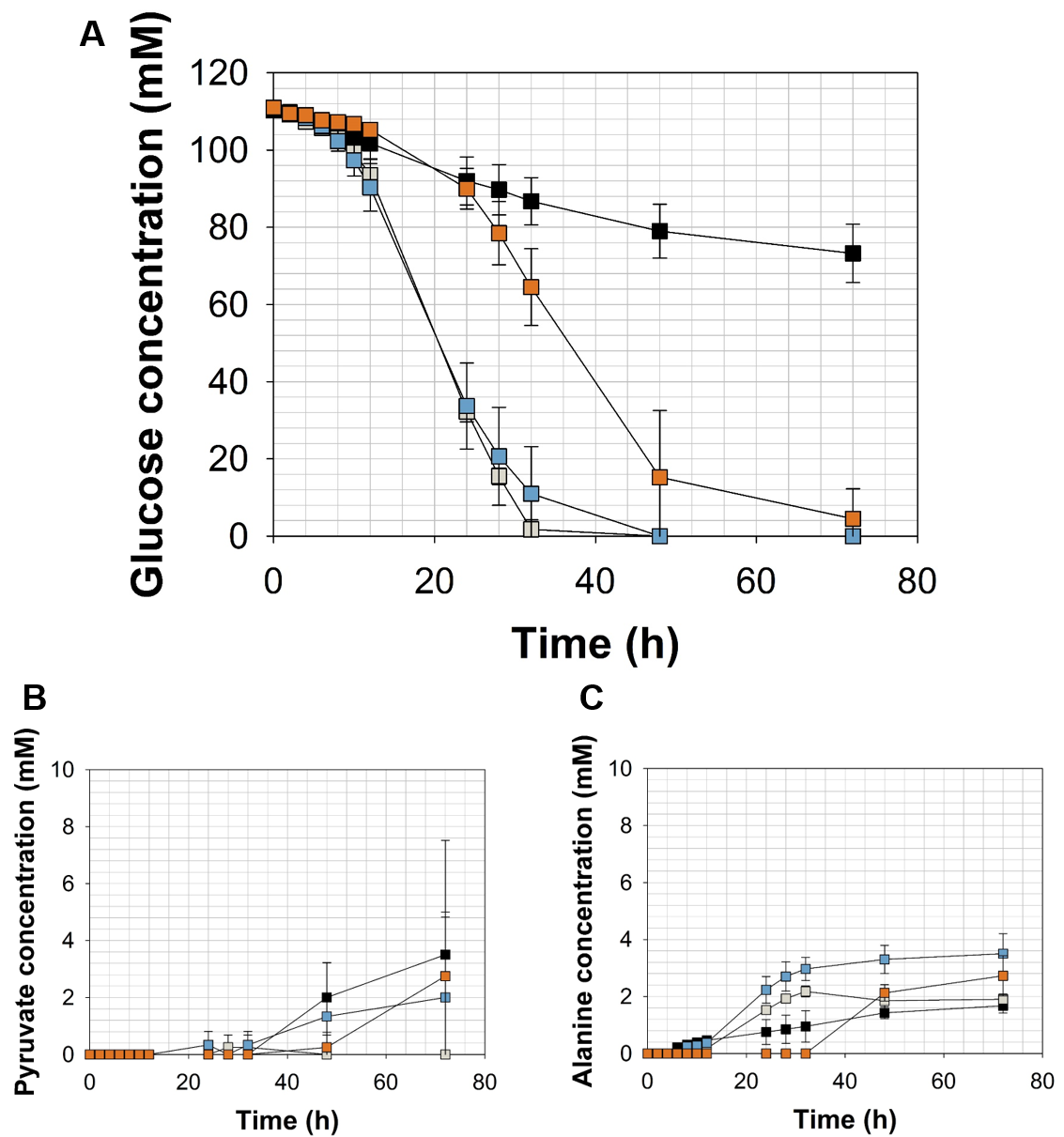


Figure S4: (A) Glucose consumption and (B – C) pyruvate and alanine accumulation of *C. glutamicum* ΔP*_aceE_*::*vanR*-P*_vanABK_**(pJC4-*ilvBNCE*) in shaking flasks with CGXII minimal medium (with 20 g ammonium sulfate L^-1^) and 20 g glucose L^-1^ without (black) and with 5 mM ferulic acid (grey), 1 mM vanillin (blue) or 7.5 mM vanillic acid (orange). Error bars represent standard deviation of cultivations of at least three biological replicates.

# Consumption of ferulic acid, vanillin or vanillic acid of *C. glutamicum* ΔP*_aceE_*::*vanR*-P*_vanABK_** (pJC4-*ilvBNCE*) and *C. glutamicum* ΔP*_aceE_*::*vanR*-P*_vanABK_** (pJC4) in shaking flask cultivations


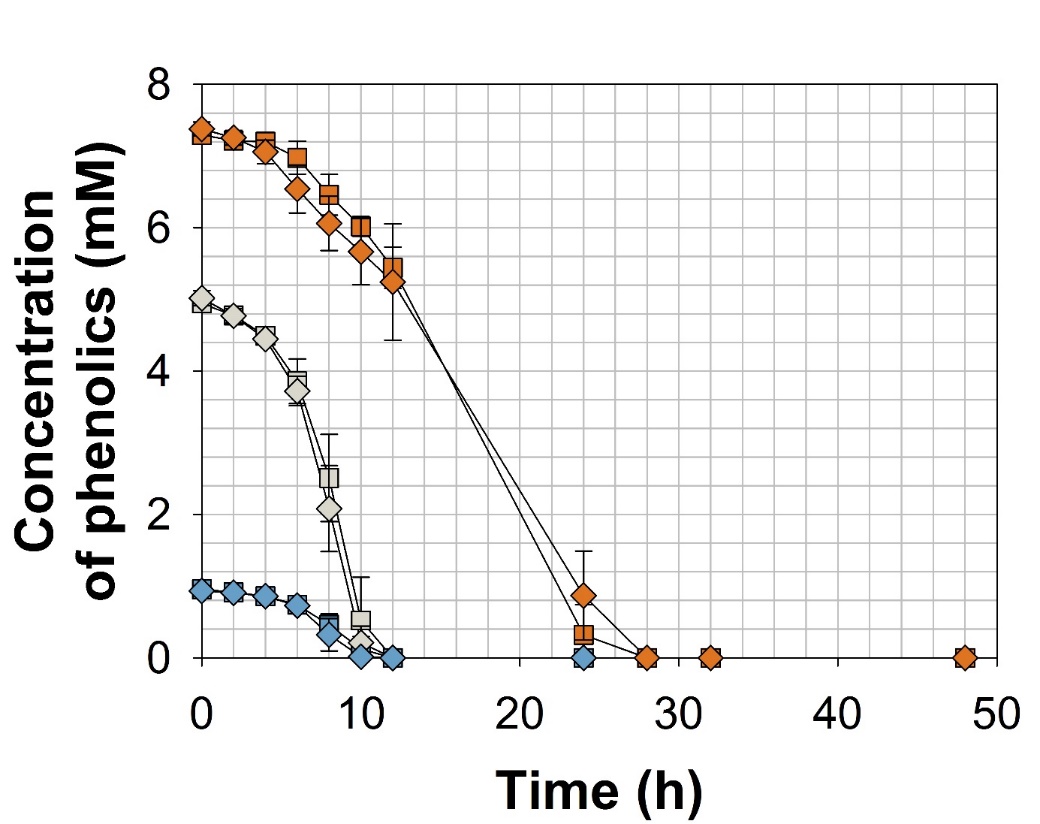


Figure S5: Phenolic compound consumption of *C. glutamicum* ΔP*_aceE_*::*vanR*-P*_vanABK_**(pJC4-*ilvBNCE*) (squares) and *C. glutamicum* ΔP*_aceE_*::*vanR*-P*_vanABK_**(pJC4) (diamonds) in shaking flasks with CGXII minimal medium (with 20 g ammonium sulfate L^-1^) and 20 g glucose L^-1^ with ferulic acid (grey), vanillin (blue) or vanillic acid (orange). Error bars represent standard deviation of cultivations of at least three biological replicates.

# Growth, consumption of glucose and accumulation of pyruvate, alanine and valine of *C. glutamicum* ΔP*_aceE_*::*vanR*-P*_vanABK_** (pJC4) in shaking flask cultivations supplemented with one specific concentration of ferulic acid, vanillin or vanillic acid


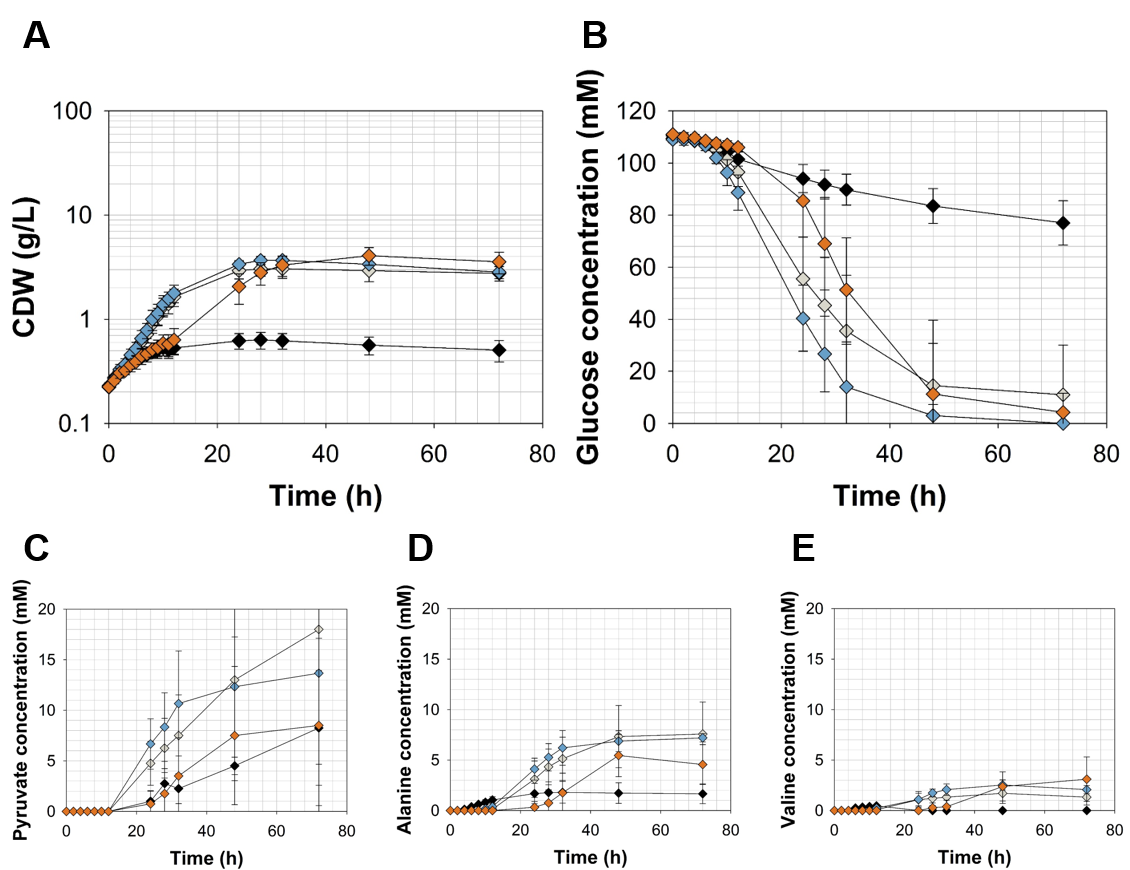


Figure S6: (A) Growth (B) glucose consumption and (C – D) pyruvate, alanine and valine accumulation of *C. glutamicum* ΔP*_aceE_*::*vanR*-P*_vanABK_**(pJC4) in shaking flasks with CGXII minimal medium (with 20 g ammonium sulfate L^-1^) and 20 g glucose L^-1^ without (black) and with 5 mM ferulic acid (grey), 1 mM vanillin (blue) or 7.5 mM vanillic acid (orange). Error bars represent standard deviation of cultivations of at least three biological replicates.
